# Supplementary material for: Importance of the 1+7 configuration of ribonucleoprotein complexes for influenza A virus genome packaging
Source: Nat Commun. 2018 Jan 4;9:54. doi: 10.1038/s41467-017-02517-w (PMC5754346; doi:10.1038/s41467-017-02517-w)

Supplementary Information

Supplementary Figure 1. Uncropped scans of blots shown in the main figures.

a. The uncropped scan of the gel shown in Fig 1b.

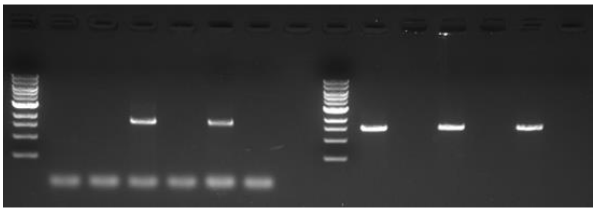

b. The uncropped scans of the blots shown in Fig 1c.

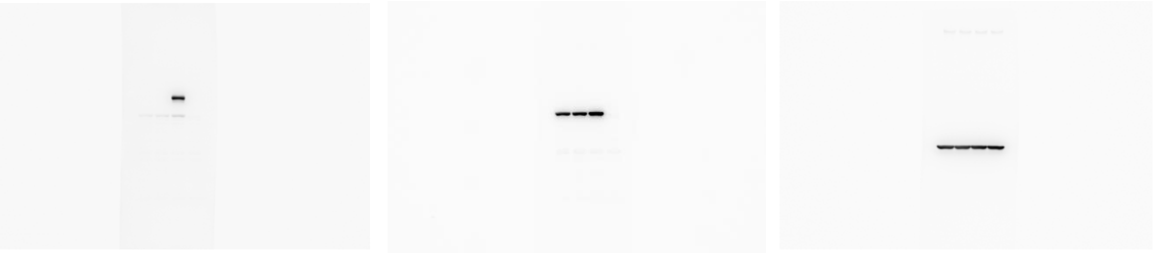

c. The uncropped scan of the gel shown in Fig 3a.

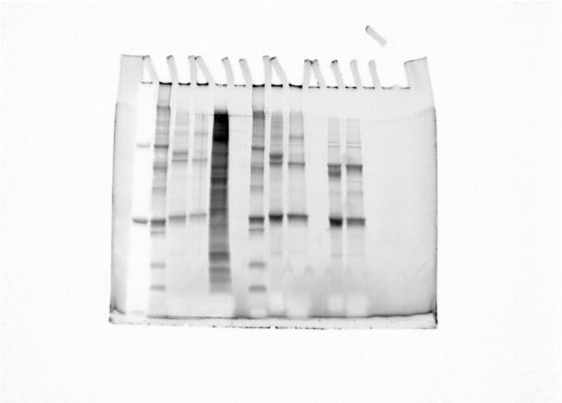

d. The uncropped scans of the blots shown in Fig 3c.

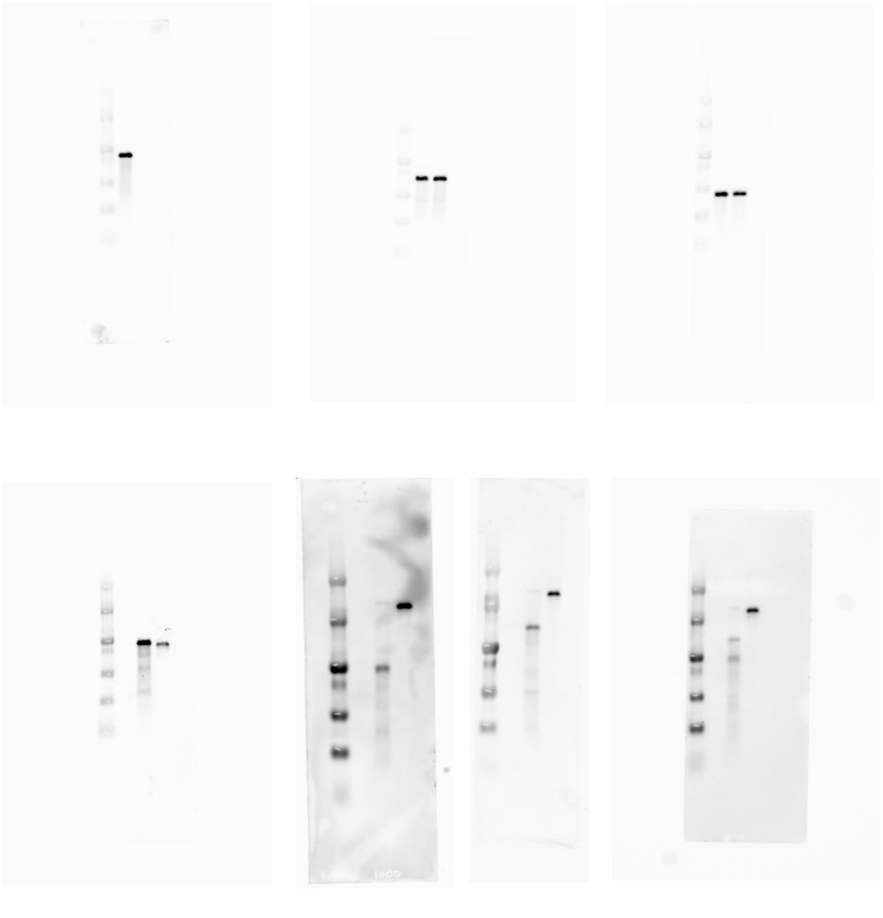

e. The uncropped scans of the blots shown in Fig 3d.

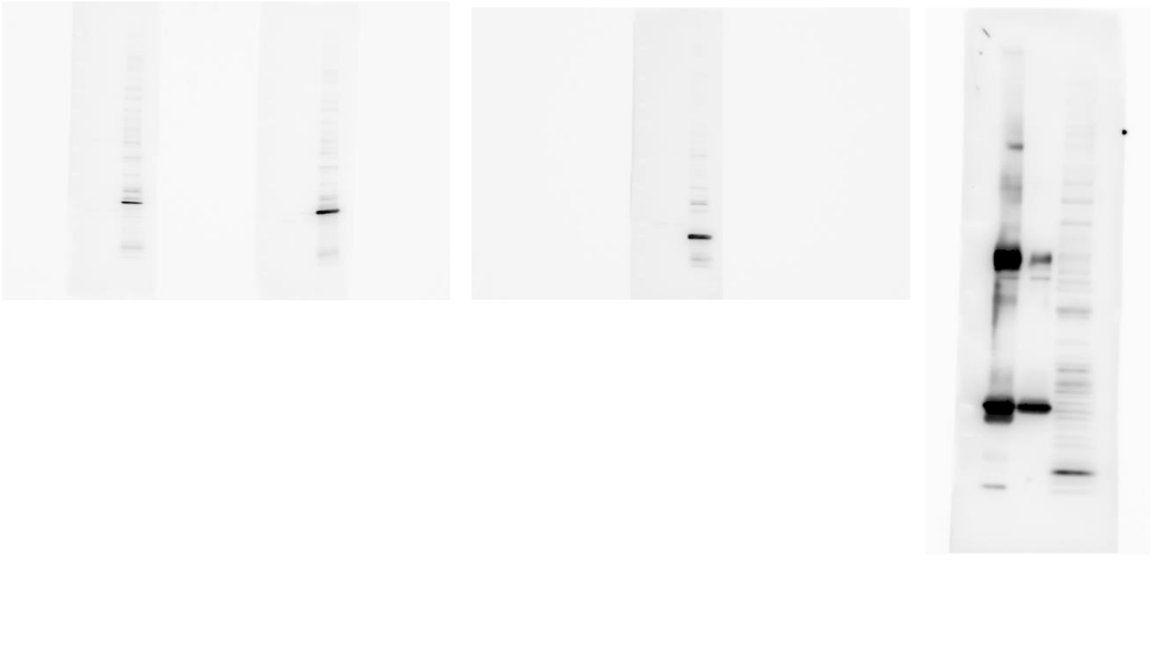

f. The uncropped scan of the blot shown in Fig 4c and d.

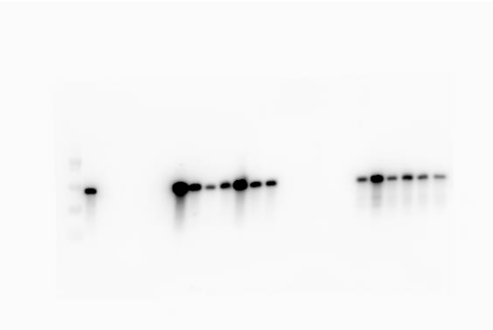

g. The uncropped scans of the blots shown in Fig 4e and f.

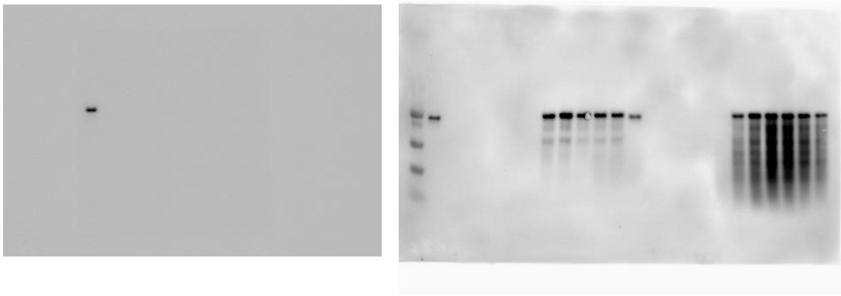

h. The uncropped scans of the blots shown in Fig 4g and h.

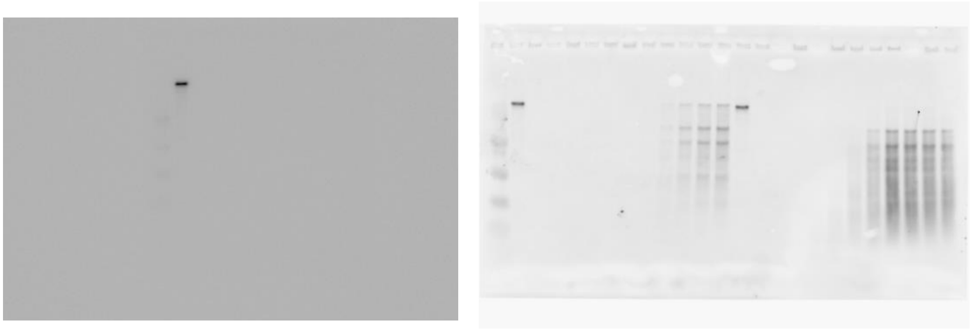

i. The uncropped scan of the blot shown in Fig 5a.

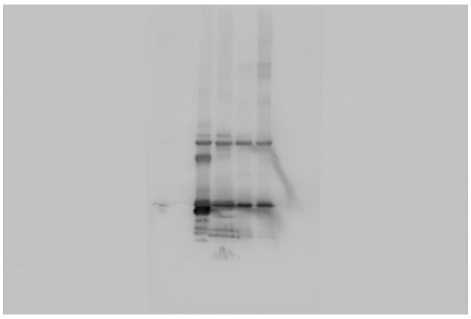

j. The uncropped scan of the blot shown in Fig 5b.

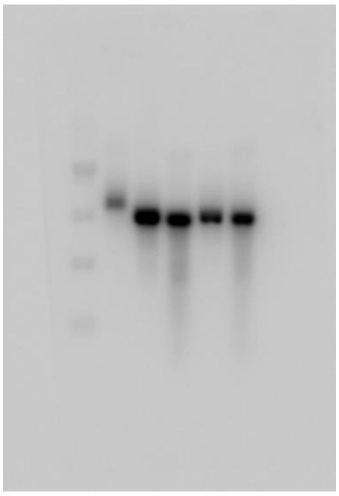

k. The uncropped scan of the blot shown in Fig 5c.

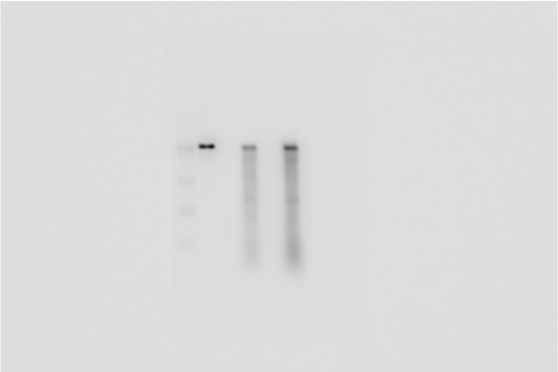

l. The uncropped scan of the blot shown in Fig 5d.

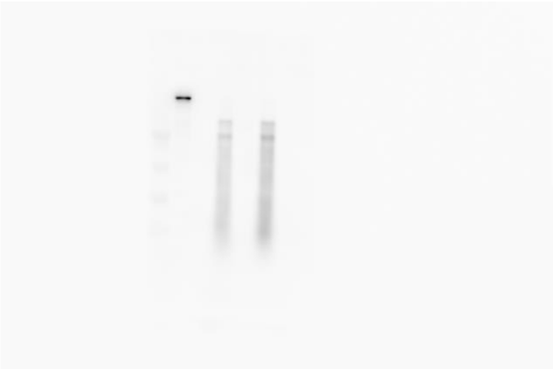

Supplement: Supplementary file 1 — Supplementary Information [file 41467_2017_2517_MOESM1_ESM.pdf]
