# Supplementary material for: Importance of the 1+7 configuration of ribonucleoprotein complexes for influenza A virus genome packaging
Source: Nat Commun. 2018 Jan 4;9:54. doi: 10.1038/s41467-017-02517-w (PMC5754346; doi:10.1038/s41467-017-02517-w)
Supplement: Supplementary file 3 — Description of Additional Supplementary Files [file 41467_2017_2517_MOESM3_ESM.pdf]

File Name: Supplementary Movie 1

Description: Movie of the 3D-reconstructed virion containing eight RNPs shown in Figure 2e.

File Name: Supplementary Movie 2

Description: Movie of the 3D-reconstructed virion containing seven RNPs shown in Figure 2f.
